# A comparative integrated gene-based linkage and locus ordering by linkage disequilibrium map for the Pacific white shrimp, *Litopenaeus vannamei*

**DOI:** 10.1038/s41598-017-10515-7

**Published:** 2017-09-04

**Authors:** David B. Jones, Dean R. Jerry, Mehar S. Khatkar, Herman W. Raadsma, Hein van der Steen, Jeffrey Prochaska, Sylvain Forêt, Kyall R. Zenger

**Affiliations:** 1 Centre for Sustainable Tropical Fisheries & Aquaculture, and the College of Science and Engineering, James Cook University, Townsville, QLD Australia; 2 ARC Hub for Advanced Prawn Breeding, James Cook University, Townsville, QLD Australia; 3 Sydney School of Veterinary Science, The University of Sydney, Camden, NSW Australia; 4 Global Gen, Desa Cikiwul Bantar Gebang Bekasi, Bekasi, Indonesia; 5 ARC Centre of Excellence for Coral Reef Studies, James Cook University, Townsville, Queensland Australia; 6 Amity Aquaculture, LLC, Cheyenne, WY USA

## Abstract

The Pacific whiteleg shrimp, *Litopenaeus vannamei*, is the most farmed aquaculture species worldwide with global production exceeding 3 million tonnes annually. *Litopenaeus vannamei* has been the focus of many selective breeding programs aiming to improve growth and disease resistance. However, these have been based primarily on phenotypic measurements and omit potential gains by integrating genetic selection into existing breeding programs. Such integration of genetic information has been hindered by the limited available genomic resources, background genetic parameters and knowledge on the genetic architecture of commercial traits for *L*. *vannamei*. This study describes the development of a comprehensive set of genomic gene-based resources including the identification and validation of 234,452 putative single nucleotide polymorphisms *in-silico*, of which 8,967 high value SNPs were incorporated into a commercially available Illumina Infinium ShrimpLD-24 v1.0 genotyping array. A framework genetic linkage map was constructed and combined with locus ordering by disequilibrium methodology to generate an integrated genetic map containing 4,817 SNPs, which spanned a total of 4552.5 cM and covered an estimated 98.12% of the genome. These gene-based genomic resources will not only be valuable for identifying regions underlying important *L*. *vannamei* traits, but also as a foundational resource in comparative and genome assembly activities.

## Introduction

Breeding programs for animal production species have traditionally been developed around phenotypic selection in conjunction with quantitative genetic theory. As with other realms of biology, animal production science is currently in the midst of a genomics revolution and there has been an increasing global focus on the development of genomic resources and subsequent identification of markers linked to genes of economic importance. Although still in its infancy as a production industry, aquaculture is perfectly situated to uptake recent advances in quantitative genetics and to integrate new genomic technologies into future breeding program designs^1^.

The globally important whiteleg shrimp or Pacific white shrimp, *Litopenaeus vannamei*, is an aquaculture species that would benefit substantially from the integration of genomic information into traditional breeding programs, particularly for disease resistance and other difficult to measure or low heritability traits. Unfortunately, even though several genetic linkage maps have been produced^2, 3^, comprehensive genomic information available for *L*. *vannamei* is still very limited and there is currently a poor understanding of fine scale genome structure and the genetic basis underlying complex commercially important traits. For example, current breeding programs for *L*. *vannamei* use traditional phenotypic selection to produce shrimp with improved growth and resistance to various viral pathogens like Taura syndrome virus (TSV)^4, 5^. While this traditional approach has been moderately successful in producing more productive shrimp strains, genetic progress using multi-trait phenotypic selection in *L*. *vannamei* has been significantly impeded by unfavourable genetic correlations between growth and disease resistance^4, 6^, as well as a poor correlated response in susceptibility to multiple diseases^7–9^. In light of these unfavourable genetic correlations between traits of interest in *L*. *vannamei* (i.e. growth and disease), breeding strategies would benefit from the integration of genetic markers tightly associated with trait variation (i.e. quantitative trait loci - QTL). The development of single nucleotide polymorphism (SNP) marker panels with the power to simultaneously identify genome-wide QTLs for complex and/or correlated traits would assist shrimp breeding strategies, as it would allow for the improved identification of selection candidates possessing advantageous genes. This would negate the current requirement for multiple selection lines and allow selection decisions for traits to be made directly on candidates, thereby increasing the accuracy of selection and resultant genetic gains. Despite recent increased research effort into *L*. *vannamei* genomics which have yielded SNP markers and moderate density linkage maps^2, 3, 10–14^, limited gene-based (Type-I) genomic resources are publically available, and production trait architecture and localisations are based on low density maps containing either AFLPs or microsatellites^12, 14, 15^. Therefore, there is still a need to develop comprehensive genome-wide SNP marker panels and dense genomic maps that allow the simultaneous detection of genome-wide QTLs for commercially important traits.

SNPs derived within expressed sequence tags (ESTs) which originate from gene coding and 3′-UTR regions are considered a valuable resource useful in linking genotype to phenotype. Anchoring EST-derived SNPs and their associated transcript sequences to a high density genomic map not only allows insights into genome structure and marker spacing across the genome, but also helps identify the biochemical pathways underlying traits of interest. This study aimed to extend on the current genomic resources available for *L*. *vannamei* by developing a gene-based commercial SNP array and genomic linkage map, demonstrate the placement of additional markers using novel locus ordering by linkage disequilibrium (LODE) methods^16, 17^, and describe the genome synteny between two commercially valuable penaeid species. The gene-based Type-I SNP marker panel and comparative genomic maps will be valuable resources for investigating genome-wide genetic trait associations, creating optimal marker sets for selective breeding and genomic prediction, understanding functional biology and genome evolution, and assisting in genome assembly.

## Methods

### Sequencing, assembly and annotation

To enable the identification and development of genome-wide Type I SNPs, total RNA was extracted from the tail muscle tissue of 30 *L*. *vannamei* individuals representing prominent domesticated industry lines (Global Gen, Indonesia), using TRIZOL® Reagent (Life Technologies). RNA from each individual were pooled together in equimolar amounts before being converted to double stranded cDNA using the Mint cDNA synthesis kit (Evrogen), and normalised using the Trimmer cDNA normalisation kit (Evrogen). Normalised cDNA was then sequenced using an Illumina GA-IIX genome analyser, which produced approximately 25 gigabases of 76 bp paired-end EST sequence data (~10× genome coverage). Illumina sequence adaptors and primers were screened and removed using the software Seqclean (https://sourceforge.net/projects/seqclean/). MOTHUR was used to remove sequences with an average quality score (Phred score) less than 15 (window size = 10 bp) and/or shorter than 50 bp in length^18^. The cleaned sequence data was assembled using Velvet V1.0^19^ and OASES^20^. Assembly parameters consisted of no extra gap penalty with all other options at default or recommended settings. Transcript assemblies were conducted at kmer lengths of k39, k41, k43, k45, k47, k49, k51 and k53 before being clustered together at a 90% sequence identify threshold using the software CD-HIT^21^. Where multiple transcript sequences were identified, only the longest sequence was retained. Transcript redundancy removal was undertaken, since it is a requirement for SNP discovery.

### Sequence annotation of Gene Ontology terms

Annotation of the assembled sequence database was achieved using a Blastx search algorithm^22^ and the NCBI non-redundant protein database conducted through the software package Blast2GO^23^. Where multiple annotations were returned, the one with the best bit score was retained. For each successfully annotated contig, gene ontology (GO) terms InterPro scan results were retrieved using Blast2GO.

### SNP discovery and filtering

To ensure high-quality SNPs were produced, strict data integrity measures were implemented. Genome-wide SNPs were identified using stringent SNP discovery filtering within the software package SAMTOOLs^24^ and custom scripts. NOVACRAFT (Novocraft Technologies, Selangor, Malaysia) was used to align the cleaned sequence reads to the full sequence assembly. The SAMTOOLs pileup command was used to produce mapping qualities. The varFilter option in SAMTOOLs was employed to filter SNPs, keeping only the most informative [i.e. minimum minor allele frequency (MAF) of 0.25, a minimum read depth of 10 reads, a minimum of two minor allele reads, a minimum SNP mapping quality of 25, a minimum flanking sequence quality of 25]. Any SNP identified within 50 bp of a candidate SNP was excluded to ensure a conservative flanking region for probe design. In addition, multi-allelic SNPs and SNPs requiring type I Illumina Infinium Probes (A/T or C/G) were removed and sequence repeat elements were masked. The resultant SNPs with the highest MAF and read depth were prioritised and submitted for assay development analysis using Illumina’s Assay Design Tool (ADT). Any SNP that returned an ADT score of less than 0.7 was excluded from the array. To ensure no unintentional duplicate SNPs were included on the array, probes for each SNP were mapped to the initial assembly using NOVOCRAFT (Novocraft Technologies, Selangor, Malaysia) and only the probes that mapped uniquely were included in the array. Following this procedure, 8,967 SNPs (8,616 novel SNPs with the highest ADT score and 351 from the public domain including those mapped in Du *et al*.^3^ and Ciobanu *et al*.^11^) were incorporated into the Illumina Infinium ShrimpLD-24 v1.0 SNP genotyping array (Table 1 and Supplementary Table S1).

**Table 1:**
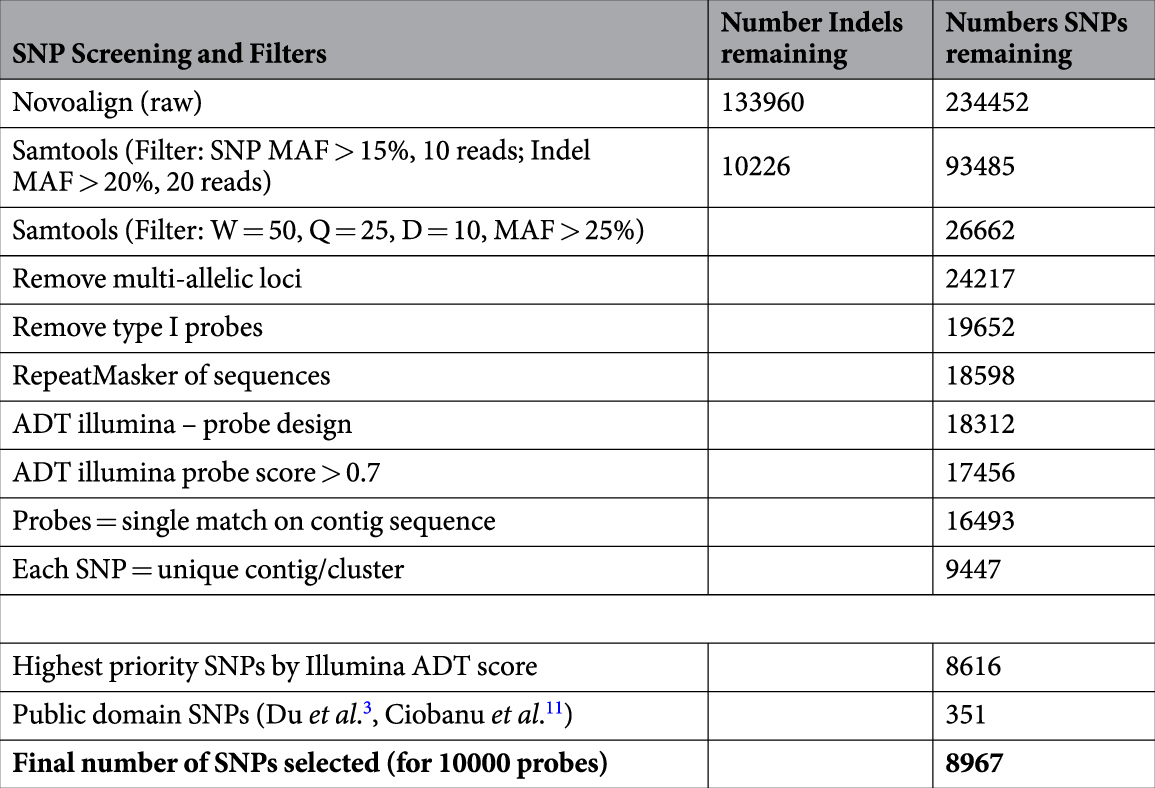
The number of SNPs retained throughout subsequent filtering and data integrity during design of the custom *L*. *vannamei* 10 k iSelect beadchip.

### Infinium array genotyping

To validate the performance of the *L*. *vannamei* Illumina Infinium ShrimpLD-24 v1.0 beadchip, 2,004 samples were genotyped, including 1,134 female and 193 male broodstock that produced families, along with 677 nauplii DNA pools (pools of >300 nauplii larvae from an individual family). For some nauplii pools, one of the two parents was either unknown or not sampled. Consequently for these families, the full unknown parental genotype was reconstructed using methods described in Supplementary Methods and Peiris, *et al*.^25^. All families were raised indoors in a Nucleus Breeding Centre under biosecure conditions from founding individuals representing most of the prominent industry domesticated/selected lines. To ensure all genotypes calls were genuine and to identify aberrant SNPs and DNA samples, strict genotypic data integrity was undertaken in GenomeStudio V2011.1 following methods outlined in Jones, *et al*.^26^. Family groups were reconstructed using SNP genotypic data (as described below) to enable the assessment of Mendelian inheritance (MI) of alleles. Genotype reproducibility between batches and across arrays was tested using 52 replicate samples and 26 replicate SNPs.

Genomic DNA was extracted either from the 2,004 *L*. *vannamei* samples or pools using a modified CTAB protocol^27^. DNA was standardised to 50 ng/µl using PicoGreen dsDNA quantification (Invitrogen), while DNA quality was inspected by agarose gel electrophoresis. All array genotyping was undertaken at PathWest Medical Laboratories, Perth, Western Australia, following manufacturer instructions^28^. Genotypes were calculated within the genotyping module of Genome Studio V2011.1 (Illumina Inc.) using the GenTrain genotype clustering algorithm. A minimum GenCall (GC) score cut-off (quality metric for each genotype) of 0.15 was used in SNP genotype clustering. The proportion of loci that produced a genotype for a sample is the sample genotyping rate. The SNP conversion rate is defined as the number of SNPs that produced a genotype divided by the number of total SNPs included. SNP validation rates were calculated as the number of SNPs with a heterozygous call divided by the number of SNPs that produced a genotype. SNPs with a minor allele frequency of greater than 0.01 were considered polymorphic. Mendelian errors for each SNP were reported as in Mendelian agreement whereby;

1 $$Mendelian\,agreement=1-(\frac{No.\,Mendelian\,errors}{No.\,loci\,genotyped})$$

All GenCall scores are reported as the 10^th^ percentile of the GC scores (GC10 scores). All SNPs were investigated for conformation to expected Hardy-Weinberg Equilibrium (HWE) and Mendelian Inheritance (MI) patterns. All recorded pedigree information was validated in a number of subsequent iterations using the 1,800 highly reliable “first class” SNPs produced from the array and the parentage programs Cervus 3.0^29, 30^ and COLONY^31^. Briefly, all individually genotyped females and male family relationships were confirmed using this integrated approach, whereby all maternal assignments were verified in COLONY (1,121), before being used to verify paternal assignments (750). Then using all validated parental relationships, COLONY was used to cluster pedigrees as an extra level of validation and to estimate unknown parents by inferring genotypes (N = 30). Any disagreements or pedigree alterations were resolved.

### Linkage mapping families and map construction

After parental relationship validation and genotype reconstruction, a total of 631 progeny from 30 grandmaternal and 19 grandpaternal traced families were selected for linkage map construction (the number of progeny within a family ranged from 8 to 33; Supplementary Fig. S2 and Supplementary Table S3). The genotypic data of these individuals over all 6,379 high quality SNPs (as described below) was manually phased into hexadecimal encoding using custom scripts and linkage analysis was conducted in Carthagene V1.3^32, 33^. Markers were segregated into linkage groups by the *group* function at a logarithm of odds (LOD) threshold of 10 and a distance threshold of 30 cM. Linkage groups were defined as groups of at least three markers ordered on a map at a LOD 3 threshold, and having agreement with independent linkage disequilibrium (LD) and LODE mapping assignment (as described below). The remaining 1,447 markers which did not have three markers ordered at a LOD 3 threshold and/or were not confirmed by LD and LODE analysis were designated as orphan markers. The defined linkage groups were subsequently constructed using a hierarchical approach whereby ordering was determined using consecutive thresholds of LOD3, LOD2 and the most likely marker position. For each consecutive threshold, maps were created using the *buildfw* function, followed by *annealing*, *flips 6* and *polish*, until the best sex average map was produced. After all linkage groups were ordered, orphan markers were tested again using two-point to determine whether they could be inserted into any ordered linkage groups. In addition, the five distal markers from both ends of each linkage group were compared by two-point to identify if any linkage groups could be merged together. Sex specific maps were also produced by locking in the sex average marker order and re-calculating interval distances based on separate male and female informative recombination events. The Kosambi^34^ mapping function was used for all centi-Morgan (cM) calculations.

### Sex- and family- specific recombination heterogeneity

To investigate sex-specific heterogeneity throughout independent linkage groups, the following goodness of fit heterogeneity test was utilised with one degree of freedom as described in Ott^35^ and Jones, *et al*.^36^;

2 $${{\rm{{\rm X}}}}^{2}=2\times \,\mathrm{ln}(10)[Z({\hat{\theta }}_{m},{\hat{\theta }}_{f})-Z(\hat{\theta },\hat{\theta })]$$

where, $Z({\hat{\theta }}_{m},{\hat{\theta }}_{f})$ is the joint sex-specific recombination rate and $Z(\hat{\theta },\hat{\theta })$ represents the recombination rate when equal male and female recombination fractions are assumed. For each test, a false discovery rate (FDR) correction was applied to correct for multiple comparisons and minimise false positives^37^.

To detect any differences in sex-specific recombination rates, ratios of female-to-male map distances were calculated (*R* = *X*_*f*_/*X*_*m*_) for each interval and linkage group as well as over the entire map. To ensure any observed sex-specific recombination was truly due to differences between the sexes, and not affected by variation in individual F1 parents, family specific heterogeneity was investigated for each F1 parent independently. LINKMFEX version 2.4^38^ was used to calculate the recombination fraction, number of co-informative meiotic events (N) and the number of recombinations (r) for all mapped locus intervals for the maternal and paternal lines of each family separately. The Zmax score (LOD) was calculated for the mother and father in each family, and combined across all mothers and fathers respectively using methods outlined in Ott^35^. The following M-test was employed to investigate individual F1 recombination heterogeneity within each mapping family Ott^35^.

3 $${{\rm{{\rm X}}}}^{2}=2\times \,\mathrm{ln}(10)[\sum {Z}_{i}({\hat{\theta }}_{i})-Z(\hat{\theta })]$$

Here, ${Z}_{i}({\hat{\theta }}_{i})$ represents the LOD scores maximum likelihood estimation (MLE) for the *i*th F_1_ reference family for a pair of markers, with $Z(\hat{\theta })$ being the total LOD score MLE of all *i*th reference families.

### Segregation distortion

Segregation distortion was investigated to determine if there was any evidence of deviations from expected Mendelian Inheritance (MI) patterns. This was investigated using log-likelihood ratio tests for goodness of fit to Mendelian expectations on manually phased genotypic data across all markers from all dams and sires as described in Sokal and Rohlf ^39^ and Jones, *et al*.^36^.

### The extent of linkage disequilibrium and integration of LODE-placed markers

Locus ordering by disequilibrium (LODE) is a novel methodology that allows the utilisation of additional linkage disequilibrium data to place unpositioned or orphan SNPs within genetic maps or scaffolds^16, 17, 40, 41^. The LODE procedure used in this study is an adaptation of the two step procedure described in Khatkar, *et al*.^16^. Firstly, SNPs are assigned to a chromosome or linkage group, then subsequently its position within this linkage group is estimated. Both of these steps rely on pair-wise estimates of linkage disequilibrium (LD). LD estimates (*r* ^*2*^ and *D*′) were computed among 6,379 SNPs and 1,963 individuals (631 individuals from mapping families, and 1,332 individuals representing prominent industry lines) using GOLD software^42^. The extent of LD among SNPs, within and across the linkage groups, was estimated using position of SNPs on the current linkage map. Placements of orphan SNPs using the LODE method were defined based on at least three pairs on a chromosome with r-squared 0.1 or more, but also looking at the maximum LD score.

### Genome coverage

Genome coverage of the integrated linkage and LODE sex-average map was calculated using observed and expected genome lengths. The observed genome length (Goa) was calculated by adding the observed linkage group lengths. The expected genome length (Ge) was produced by multiplying the length (cM) of each linkage group by (*m* + *1*)/(*m* − *1*), where *m* is the number of loci in each linkage group^43^. The total expected genome length was then the sum of Ge from all linkage groups. Genome coverage (Coa), was calculated by dividing Goa by Ge^44^.

### Comparative genome analysis

Syntenic relationships were explored for the integrated linkage and LODE map against three previously published maps, a *L*. *vannamei* SNP linkage map with 6,359 markers^2^, a *L*. *vannamei* SNP linkage map with 418 SNP markers^3^, and a *Penaeus monodon* linkage map with 3,959 SNPs^45^. Assignment of orthologous sequences were undertaken by reciprocal BLAST searches of contigs sequences from which SNPs were discovered in the present study against respective sequence databases available for the maps of Yu, *et al*.^2^, Du, *et al*.^3^ and Baranski, *et al*.^45^ (at an e-value threshold of >1e-5). Comparisons to Yu, *et al*.^2^ were undertaken using their contiguous sequences generated from their genome survey sequencing, bacterial artificial chromosomes (BACs) and marker sequences, whereas comparisons to Baranski, *et al*.^45^ were undertaken with the contig sequences associated with their mapped SNPs. The primary hit was retained in each case. In addition to sequence similarity search of the marker sequences published in Du, *et al*.^3^, 159 SNPs from a previously published low density SNP map^3^ were included on our genotyping array to allow the direct comparison of their linkage map to ours. Comparison of genome synteny in this case was undertaken by matching marker IDs of all SNPs from this current study that were directly genotyped and mapped with our integrated map. BLAST annotations to *Daphnia pulex* and *Drosophila melanogaster* for SNPs with common IDs were also carried across from Du, *et al*.^3^. Oxford Grids^46^ of the integrated map presented here versus Yu, *et al*.^2^, Du, *et al*.^3^ and Baranski, *et al*.^45^ were plotted using custom R scripts to confirm mapping position and illustrate genome synteny. An example linkage group (LG4) was drawn using ArkMap^47^ to illustrate genome conservation.

### Data availability

The assembled contig sequences and mapped raw reads generated within the current study have been submitted to GenBank as a SRA database (Accession number: SRP094129). All SNPs included on the Illumina Infinium ShrimpLD-24 v1.0 array have been submitted to dbSNP on NCBI [Accession numbers: ss2137297825–ss2137306471 from the current study; rs159816077–rs159831399 mapped in Du *et al*.^3^; and rs142459135–rs142459627 developed in Ciobanu *et al*.^11^]. The Illumina Infinium ShrimpLD-24 v1.0 array is available from https://www.illumina.com/products/by-type/microarray-kits/infinium-shrimp-ld.html. All remaining data used and/or analysed during the current study are available from the corresponding author on reasonable request.

## Results

### Sequencing and assembly of transcripts

In total, over 25 Gb of sequence data (329 million raw EST sequences, 76 bp paired-end, ~10× genome coverage) was produced from an Illumina GA-IIx run. After clean-up and trimming, 19.7 Gb of sequence data was retained (average Phred score of 25.9). Assembly of the cleaned-up sequence data (including transcript redundancy removal) produced 76,963 contigs. The N50 of the assembly was 2,375 bp, the average contig length was 1,429 bp and median contig length was 955. Over 72% of the 76,963 contigs had a read depth coverage of greater than 50 reads (average read depth over all contigs was 2527.5 reads). The assembled contig sequences and mapped raw reads have been submitted to GenBank as a SRA database (Accession number: SRP094129). This is a significant genomic resource enabling the sequence data mining of 27,477 specific genes (see below) and *in-silico* detection of over 234,452 SNPs and 133,960 indels (Table 1).

### Sequence annotation and gene ontology terms

**Figure 1:**
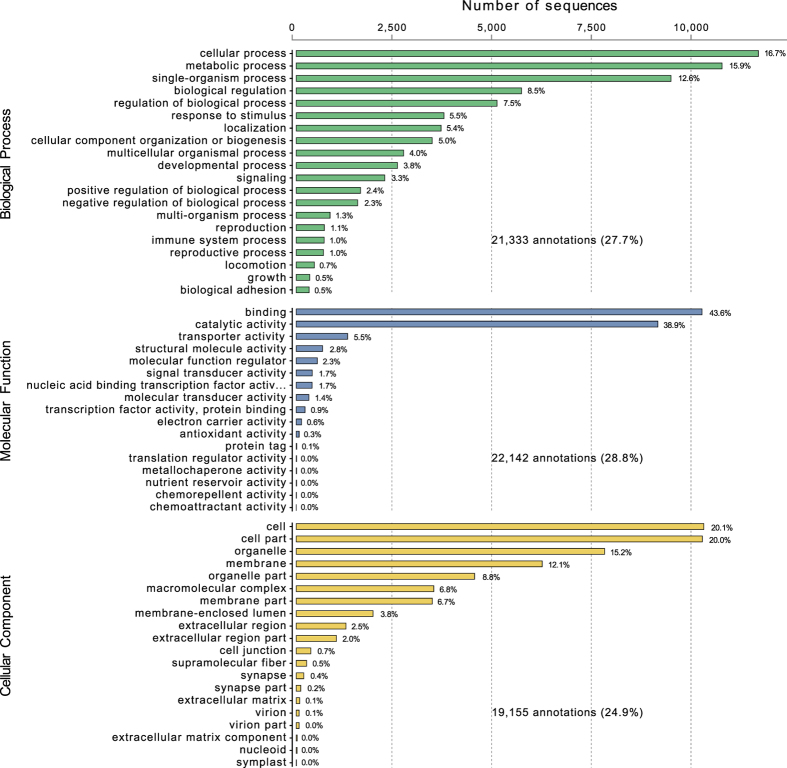
Proportions of Gene Ontology (GO) annotations of the assembled 454 mantle tissue transcripts from *Litopenaeus vannamei*.

Blastx searches against NCBI’s non-redundant protein database produced 30,317 hits from the 76,963 contigs. Of these sequences, 27,477 (24.7%) also had GO categories assigned, from which these genes were categorised into biological processes (21,333), molecular function (22,142) and cellular components (19,155) (Fig. 1 and Supplementary Table S4). Within the listed biological processes, most genes were involved in cellular and metabolic processes (32.7%). The most common molecular function designations were binding (43.6%) and catalytic activity (38.9%). Finally, cell (20.1%), cell part (20.0%) and organelle (15.2%) formed the most common GO terms within cellular component designations. A total of 12,957 unique gene hits were identified including Myosin and Myostatin/Growth Differentiation Factor-11, which are involved in muscle cell growth^48, 49^, as well as genes involved in immune response pathways such as apoptosis, MAPK signalling, toll-like receptor and antigen processing and presentation^50^.

### SNP discovery and development of commercial array

#### In-silico SNP discovery and filtering

From the assembled sequence dataset, 234,452 putative SNPs and 133,960 indels were identified *in-silico* before strict filtering parameters were applied. By filtering out all SNPs with a read depth less than 10 reads and a minor allele frequency (MAF) of less than 0.25, a total of 26,662 high-quality SNPs were identified. A further 2,445 multi-allelic SNPs, 4,565 SNPs requiring Type I Illumina Infinium probes and 1,054 highly repetitive SNP probes were removed before ADT analysis. Illumina’s ADT analysis calculates the effectiveness of the SNP probes on the array. A total of 1,142 SNPs did not return ADT values > 0.7 and 1,006 SNPs did not map to unique contigs and were removed. A further 7,003 SNPs were excluded due to being located within the flanking region of another SNP resulting in a final list of 9,447 SNPs. Of these, 8,967 SNPs (8,616 novel SNPs with the highest ADT score and 351 from the public domain including those developed in Ciobanu, *et al*.^11^ and mapped in Du, *et al*.^3^) were incorporated into the Illumina Infinium ShrimpLD-24 v1.0 SNP genotyping array enabling high throughput, cost effective and accurate genotyping (Table 1 and Supplementary Table S1). The average MAF and ADT score of these high-value SNPs was 37% and 0.95 respectively. All SNPs included on the Illumina Infinium ShrimpLD-24 v1.0 array have been submitted to dbSNP on NCBI (Accession numbers: ss2137297825 - ss2137306471 from the current study; rs159816077 - rs159831399 mapped in Du, *et al*.^3^; and rs142459135 - rs142459627 developed in Ciobanu, *et al*.^11^). The Illumina Infinium ShrimpLD-24 v1.0 array is available from https://www.illumina.com/products/by-type/microarray-kits/infinium-shrimp-ld.html.

#### Infinium array genotyping and validation

In total, 2,004 shrimp samples were genotyped, including 1,134 female and 193 male parents of families, along with 677 nauplii pools. From these samples, 70 individuals produced call rates of less than 90% and were subsequently removed from further analysis leaving 1,257 unique individuals to investigate SNP array performance. Analysis of the resulting genotypic data revealed that 6.01% of the SNPs did not amplify successfully (probe did not bind to the DNA) and 13.04% of the SNPs returned ambiguous clusters. From the resulting 7,259 SNPs, the SNP conversion rate was calculated to be 80.95%. Within the converted SNPs, 318 SNPs did not return heterozygous genotype calls and therefore were considered monomorphic. After the removal of the monomorphic SNPs, 6,941 remained resulting in a SNP validation rate of 95.62%. To estimate the proportion of informative or polymorphic SNPs, within this experimental population, a further 562 SNPs with deviations from HWE and MI errors were excluded, resulting in 6,379 SNPs (87.88%) with minimal errors (Table 2). Further stringent data integrity (i.e. excluding SNPs with a MAF  < 0.01, SNP duplication, or low call rates) resulted in the exclusion of an additional 323 SNPs (Table 2). From the final dataset of 6,056 high quality SNPs, the SNP call rate was extremely high (98.92%) and the Mendelian inheritance concordance exceeded 99.9%. The average minor allele frequency of these high-value SNPs was 0.37. Summary statistics for all SNPs included on the array are included in Supplementary Table S1. A total of 52 replicate samples were included to evaluate final array genotyping performance. No major deviations between replicate samples were observed, resulting in sample concordance exceeding 99.9%. This provides strong support for highly reliable genotypic data across all validated SNPs.

**Table 2:** SNP array performance indicating the number of SNPs retained over subsequent filtering procedures.

SNP Exclusion Category	#SNPs excluded	#SNPs remaining
Total Number of SNPs:		**8967**
Probe Didn’t Bind	539	
Ambiguous Clusters	1169	
Number of SNPs producing genotypes (SNP conversion rate):		**7259 (80.95%)**
Monomorphic	318	
Number Validated SNPs (SNP validation rate):		**6941 (95.62%)**
HWE deviations (Heterozygous Excess/Deficit)	163	
Mendelian Inheritance Errors	399	
Number of SNPs with minimal errors:		**6379 (87.88%)**
Mendelian Inheritance Errors (<0.01)	48	
MAF 0.01	42	
Duplicated SNPs	43	
Call rate <90%	140	
Only 2 Clusters	50	
Number of SNPs with no errors:		**6056 (83.43%)**

### Linkage map construction and LODE integration

**Figure 2:**
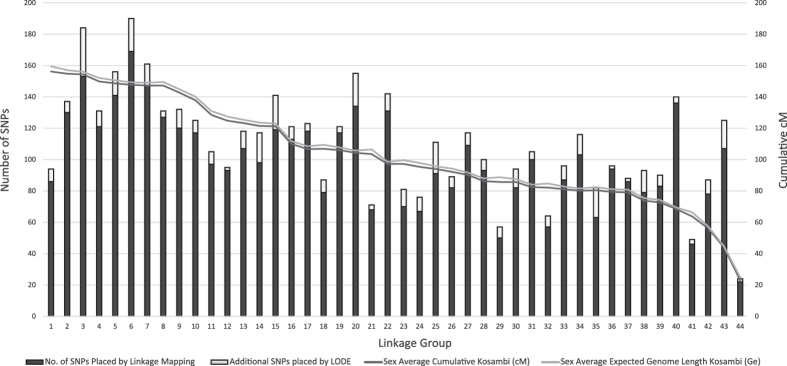
Distribution of SNPs placed using linkage and LODE mapping methods across the 44 linkage groups.

A total of 708,209 phase known informative meiotic events were utilised to place and order SNPs across linkage groups. The average number of informative events per mapped locus was 147.02 (ranging from 4 to 444) compared to an average of 28.30 informative meiotic events for unmapped markers. A total of 4,370 SNPs were successfully mapped to their most likely position within the 44 linkage groups, which spans a total of 4,552.5 cM of the estimated sex-average 4,619.3 cM genome length, covering 98.12% of the *L*. *vannamei* genome. By utilising this linkage map in LODE analysis, an additional 447 markers were placed with high confidence. This integrated map (Build 1.2) contained a total of 4,817 SNPs which reduced the average marker interval across the genome to 0.97 cM, or 2.67 when all intervals of 0 cM were excluded (Fig. 2 and Table 3 and Supplementary Table S5). Linkage groups were ordered based on their total cM length.

**Table 3:** Descriptive statistics for the *Litopenaeus vannamei* integrated linkage and LODE map (build 1.2).

Linkage group	No. SNPs placed by linkage mapping	No. SNPs placed by linkage & LODE mapping	Additional SNPs placed by LODE	Sex average map length (cM)	Female map length (cM)	Male map length (cM)	Sex average expected genome length (Ge)	Female: male ratio	Average interval (cM) (SD)	Average interval excluding 0’s (cM) (SD)	All	0–1 cM	1–2 cM	2–3 cM	>3 cM
1	86	94	8	156.10	165.00	142.10	*159*.*46*	*1*.*16*	1.66 (±3.33)	3.47 (±4.13)	45	11	9	6	19
2	130	137	7	154.80	167.70	148.40	*157*.*08*	*1*.*13*	1.13 (±2.15)	2.76 (±2.62)	56	15	14	7	20
3	153	184	31	154.30	158.90	148.10	*155*.*99*	*1*.*07*	0.84 (±2.52)	2.66 (±3.93)	58	24	17	6	11
4	121	131	10	149.80	140.70	164.30	*152*.*10*	*0*.*86*	1.14 (±2.95)	3.4 (±4.3)	44	8	12	8	16
5	141	156	15	148.60	151.70	153.70	*150*.*52*	*0*.*99*	0.95 (±1.99)	2.32 (±2.55)	64	23	18	6	17
6	169	190	21	147.70	162.80	118.90	*149*.*26*	*1*.*37*	0.78 (±2.05)	2.38 (±3.03)	62	25	18	5	14
7	147	161	14	147.20	141.60	155.80	*149*.*04*	*0*.*91*	0.91 (±1.78)	2.26 (±2.19)	65	22	20	7	16
8	127	131	4	147.20	136.50	175.90	*149*.*46*	*0*.*78*	1.12 (±1.99)	2.45 (±2.33)	60	11	23	10	16
9	120	132	12	142.70	155.30	114.20	*144*.*88*	*1*.*36*	1.08 (±2.18)	2.55 (±2.74)	56	17	14	6	19
10	117	125	8	137.90	121.10	166.60	*140*.*12*	*0*.*73*	1.1 (±2.82)	3.28 (±4.09)	42	9	9	10	14
11	97	105	8	128.50	129.30	130.80	*130*.*97*	*0*.*99*	1.22 (±3.14)	2.57 (±4.17)	50	17	18	7	8
12	93	95	2	124.80	141.20	102.10	*127*.*46*	*1*.*38*	1.31 (±2.47)	3.04 (±2.99)	41	11	11	3	16
13	107	118	11	123.30	136.20	99.70	*125*.*41*	*1*.*37*	1.04 (±2.05)	2.47 (±2.54)	50	16	14	7	13
14	98	117	19	121.50	128.70	110.30	*123*.*59*	*1*.*17*	1.04 (±2.79)	3.12 (±4.13)	39	10	10	4	15
15	119	141	22	121.20	127.70	112.20	*122*.*93*	*1*.*14*	0.86 (±2.41)	2.63 (±3.64)	46	11	20	4	11
16	113	121	8	109.90	106.10	121.00	*111*.*73*	*0*.*88*	0.91 (±2.43)	2.97 (±3.67)	37	14	7	4	12
17	118	123	5	106.70	100.20	126.60	*108*.*45*	*0*.*79*	0.87 (±1.8)	2.48 (±2.3)	43	14	8	8	13
18	79	87	8	106.90	113.90	91.10	*109*.*39*	*1*.*25*	1.23 (±2.3)	3.05 (±2.77)	35	8	10	2	15
19	117	121	4	106.00	102.60	76.90	*107*.*77*	*1*.*33*	0.88 (±1.95)	2.26 (±2.6)	47	22	7	5	13
20	134	155	21	104.30	102.10	87.30	*105*.*65*	*1*.*17*	0.67 (±1.56)	2.22 (±2.15)	47	15	12	9	11
21	68	71	3	103.50	112.70	86.10	*106*.*46*	*1*.*31*	1.46 (±2.84)	3.34 (±3.52)	31	11	3	4	13
22	131	142	11	97.20	92.60	112.40	*98*.*58*	*0*.*82*	0.68 (±2.08)	2.37 (±3.33)	41	11	15	9	6
23	70	81	11	97.20	83.40	125.70	*99*.*63*	*0*.*66*	1.2 (±2.5)	3.47 (±3.22)	28	5	6	7	10
24	67	76	9	95.30	92.20	59.70	*97*.*84*	*1*.*54*	1.25 (±2.51)	3.29 (±3.15)	29	4	9	3	13
25	91	111	20	94.00	83.40	109.70	*95*.*71*	*0*.*76*	0.85 (±2.17)	2.69 (±3.19)	35	13	8	5	9
26	82	89	7	92.20	82.70	108.10	*94*.*30*	*0*.*77*	1.04 (±2.4)	3.07 (±3.32)	30	11	7	2	10
27	109	117	8	90.20	96.30	77.80	*91*.*76*	*1*.*24*	0.77 (±1.63)	2.31 (±2.11)	39	13	7	8	11
28	93	100	7	86.20	88.50	88.90	*87*.*94*	*1*.*00*	0.86 (±2.99)	2.54 (±4.74)	34	13	8	9	4
29	50	57	7	85.70	95.80	73.40	*88*.*76*	*1*.*31*	1.5 (±3.77)	4.29 (±5.42)	20	2	7	2	9
30	82	94	12	85.70	73.90	103.60	*87*.*54*	*0*.*71*	0.91 (±2.38)	2.68 (±3.48)	32	13	6	5	8
31	100	105	5	82.40	79.60	87.00	*83*.*98*	*0*.*91*	0.78 (±1.68)	2.17 (±2.2)	38	15	9	4	10
32	57	64	7	82.10	82.40	92.70	*84*.*71*	*0*.*89*	1.28 (±2.86)	3.91 (±3.86)	21	3	4	3	11
33	87	96	9	81.10	79.90	99.80	*82*.*81*	*0*.*80*	0.84 (±2.16)	2.46 (±3.13)	33	14	5	7	7
34	103	116	13	80.10	79.50	85.10	*81*.*49*	*0*.*93*	0.69 (±1.24)	1.91 (±1.39)	42	11	14	8	9
35	63	82	19	80.40	75.30	91.50	*82*.*39*	*0*.*82*	0.98 (±2.08)	3.09 (±2.68)	26	7	7	2	10
36	94	96	2	79.40	71.60	88.10	*81*.*07*	*0*.*81*	0.83 (±2)	1.99 (±2.71)	40	19	9	4	8
37	86	88	2	79.00	71.40	69.30	*80*.*82*	*1*.*03*	0.9 (±1.5)	1.98 (±1.69)	40	11	13	8	8
38	79	93	14	73.80	82.40	73.20	*75*.*40*	*1*.*13*	0.79 (±2.46)	3.35 (±4.19)	22	5	5	6	6
39	83	90	7	72.60	64.30	90.10	*74*.*23*	*0*.*71*	0.81 (±1.83)	2.5 (±2.49)	29	9	5	9	6
40	136	140	4	68.50	69.20	70.50	*69*.*49*	*0*.*98*	0.49 (±1.52)	1.8 (±2.49)	38	21	8	3	6
41	46	49	3	63.70	69.50	51.60	*66*.*35*	*1*.*35*	1.3 (±2.73)	3.35 (±3.55)	19	6	4	3	6
42	78	87	9	56.10	56.90	55.10	*57*.*40*	*1*.*03*	0.64 (±1.35)	1.7 (±1.75)	33	15	10	2	6
43	107	125	18	43.80	41.40	48.50	*44*.*51*	*0*.*85*	0.35 (±0.82)	1 (±1.12)	44	29	9	3	3
44	22	24	2	22.90	16.40	28.40	*24*.*89*	*0*.*58*	0.95 (±1.38)	2.08 (±1.34)	11	3	2	1	5
**Total/average**	**4370**	**4817**	**447**	**4532**.**50**	**4530**.**60**	**4522**.**30**	***4619***.***32***	***1***.***02***	**0**.**97 (±2**.**22)**	**2**.**67 (±3**.**02)**	**1751**	**571**	**454**	**241**	**485**
**Genome Coverage**				**98**.**12%**											

All cM distances are in calculated using Kosambi functions.

### Sex-specific and family-specific recombination heterogeneity

Sex-specific maps were also produced using the sex-average marker order to recalculate marker intervals based on 447,640 phase-known informative meiotic events for the female map and 260,569 phased known informative meiotic events for the male map. The total lengths of the female and male maps were 4,530.60 cM and 4,522.30 cM respectively (Table 3). Minimal differences in sex-specific recombination were observed throughout the linkage groups (Supplementary Fig. S6). However, LG23 and LG44 displayed slightly larger male maps and LG6, LG9, LG12, LG13 and LG24 displayed slightly larger female maps. Overall, the average female-to-male ratio was 1.02:1. The sex-specific log10 likelihood for each linkage group, averaged between the sexes ranged from −744.740 to −190.780 (average −516.333) and the total sex-specific log10 likelihood was −22,718.645. Cumulative cM distances of the sex average, female and male maps indicate that there is no pronounced pattern of sex-specific recombination throughout the 44 linkage groups (Supplementary Fig. S6).

Investigations into family specific heterogeneity also indicate minimal significant deviations throughout all maps after false discovery rate (FDR) corrections. Only one interval on the female map (66909_123–44494_691) and six intervals on the sex average map (75525_46–34150_668, 61146_423–57343_1404, 804_148–23736_451, 23736_451–24606_1440, 66909_123–44494_691 and 7007_119–48446_2196) returned significant recombination heterogeneity after FDR (*χ* ^*2*^ = 11.809–31.301, *P* = 0.00011–0.00059, df = 1–9).

### Segregation distortion

A total of 540 significant segregation distortions were detected across all markers and families following FDR correction (Supplementary Table S7). The majority of these distortions (82.3%) were within families F1014, M1014 and F1002. As no significant family specific heterogeneity was detected for these distortions, they show no evidence of influencing calculations on mapping distances.

### Extent of linkage disequilibrium

**Figure 3:**
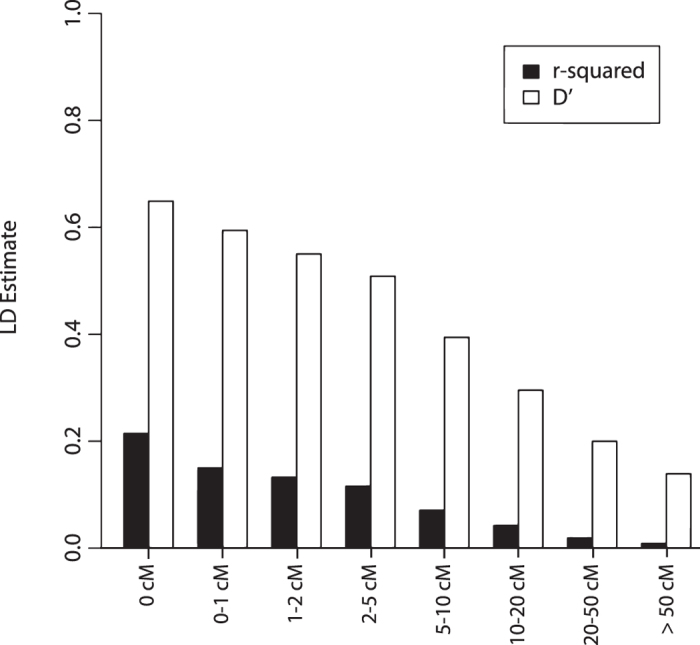
Mean linkage disequilibrium (LD) estimates at different linkage map distances throughout the *P*. *maxima* genome for *r* ^*2*^ and *D*′.

Linkage disequilibrium estimates declined gradually with increasing map intervals both throughout the genome and for each linkage group (Fig. 3 and Supplementary Table S8). This is accentuated by the relatively high mean *r* ^*2*^ values for SNP pairs less than 5 cM. Between the 4,817 adjacent SNP pairs, the mean *r* ^*2*^ estimates were 0.184 (with a median of 0.096).

### Comparative genome analysis

Sequence similarity Blastn searches of assembled contig sequences to mapping sequence data published in Yu, *et al*.^2^ and Baranski, *et al*.^45^ returned 5,591 and 2,499 hits respectively. Of these hits, a total of 67 SNPs for Yu, *et al*.^2^ and 275 SNPs for Baranski, *et al*.^45^ were in common between our integrated and previously published maps (Supplementary Table S9). The small number of blast hit results to Yu, *et al*.^2^, is most likely due to the large number of contigs from which the SNPs originated in both studies (i.e. 7.4 million contigs from Yu, *et al*.^2^ and 76,963 from our transcriptome assembly), as well as the relatively low number of mapped SNPs (6,147 for Yu, *et al*.^2^ and 4,817 from our integrated map). Between these two studies, SNPs were also developed using different methodologies. Yu, *et al*.^2^ utilised genomic DNA sequencing and specific-locus amplified fragment sequencing (SLAF-seq), whereby the SNP discovery method utilised in the current study involved transcriptome sequencing, SNP identification and design which was optimal for developing Illumina custom array probes.

Of the 159 SNPs developed in Du, *et al*.^3^ and included on our genotyping array, 83 were assigned a map position in the integrated map (Supplementary Table S9). In addition, sequence similarity searches of our mapped contig sequences (from which the mapped SNPs were designed) to the marker sequences from Du, *et al*.^3^ returned 38 hits (evalue < 0.01, similarity >95%). Of these 38 marker sequence and contig matching pairs, 30 of the respective contigs were mapped adjacent to its pair confirming its placement and blast hit (average distance = 1.08 ± 2.1 cM).

**Figure 4:**
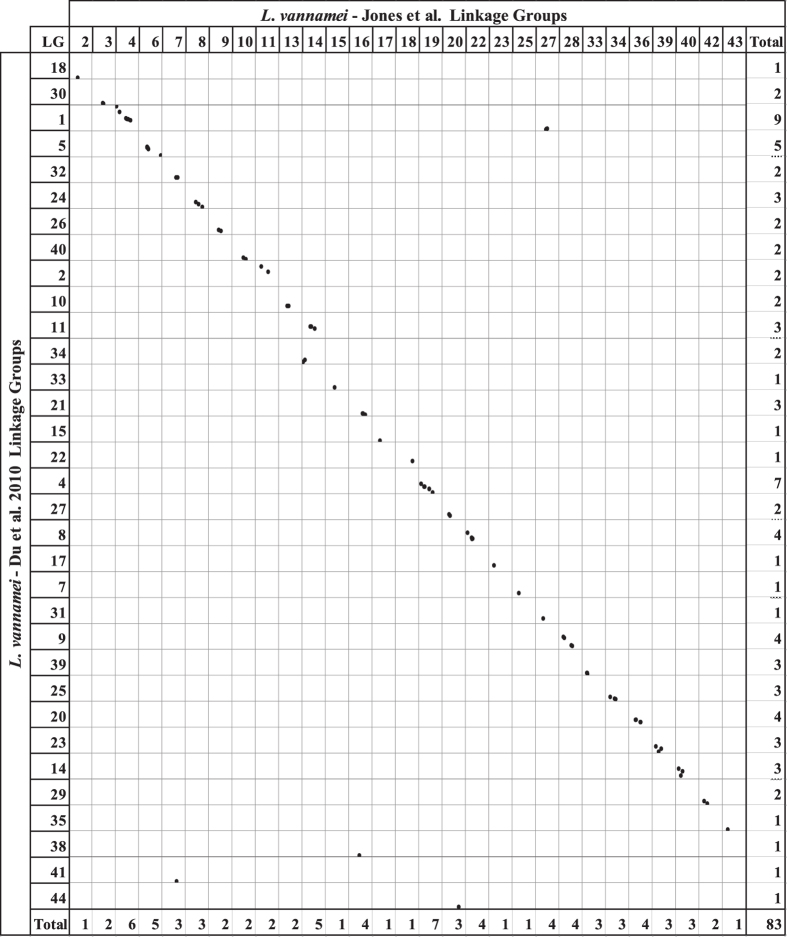
Homologous linkage map relationships between the integrated linkage and LODE *L*. *vannamei* map and the *L*. *vannamei* linkage map produced in Du, *et al*.^3^. Each dot represents a homologous locus proportional to cM lengths (Kosambi).

**Figure 5:**
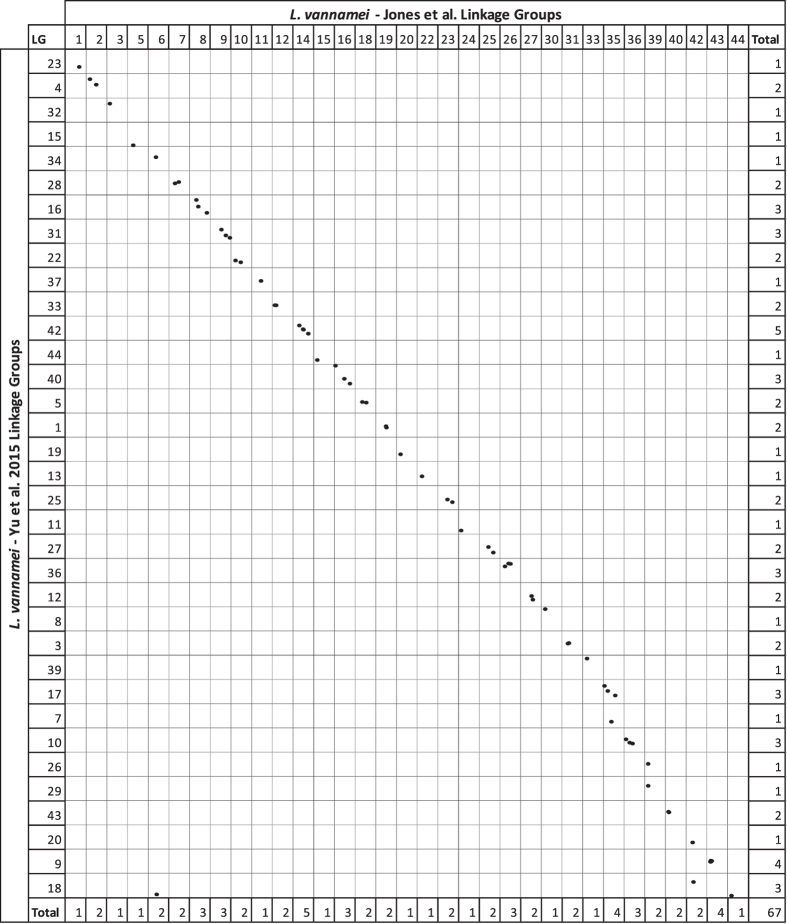
Homologous linkage map relationships between the integrated linkage and LODE *L*. *vannamei* map and the *L*. *vannamei* linkage map produced in Yu, *et al*.^2^. Each dot represents a homologous locus proportional to cM lengths (Kosambi). Data from Yu, *et al*.^2^ was utilised under a Creative Commons Attribution 4.0 International License (https://creativecommons.org/licenses/by/4.0/).

**Figure 6:**
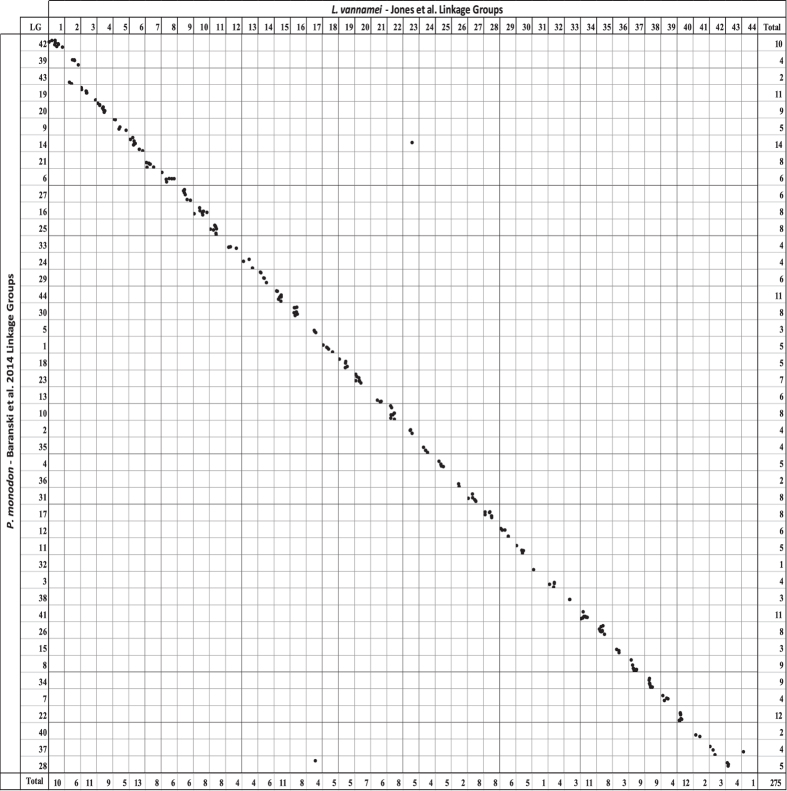
Homologous linkage map relationships between the integrated linkage and LODE *L*. *vannamei* map and the *P*. *monodon* linkage map produced in Baranski, *et al*.^45^. Each dot represents a homologous locus proportional to cM lengths (Kosambi). Data from Baranski, *et al*.^45^ was utilised under a Creative Commons Attribution 4.0 International License (https://creativecommons.org/licenses/by/4.0/).

**Figure 7:**
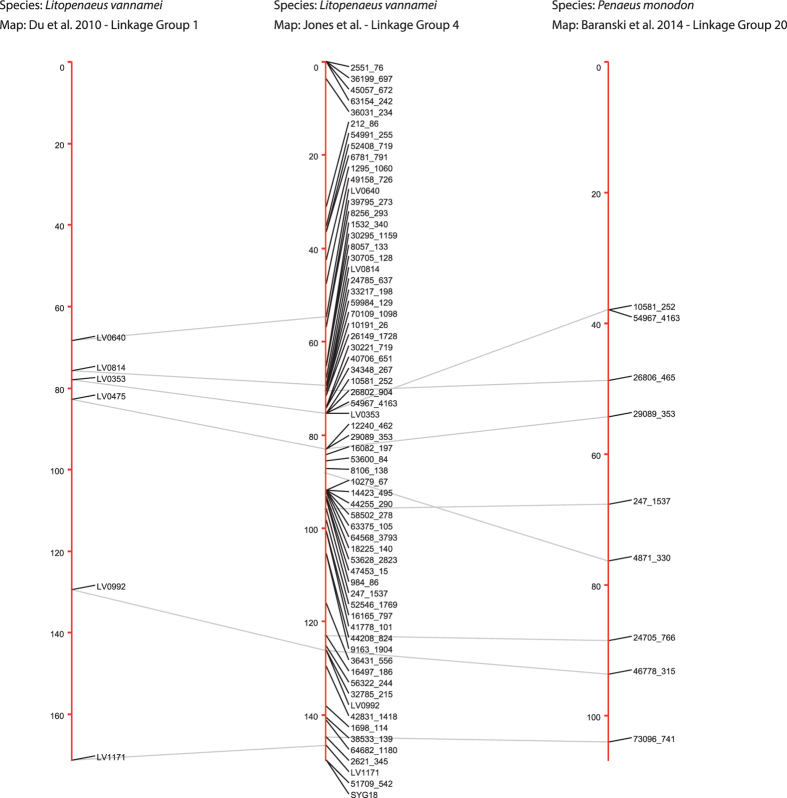
Demonstration of synteny analysis between LG4 of the integrated map, LG1 from Du, *et al*.^3^ and LG20 from Baranski, *et al*.^45^. Only matched markers are listed. Data from Baranski, *et al*.^45^ was utilised under a Creative Commons Attribution 4.0 International License (https://creativecommons.org/licenses/by/4.0/).

The identified homologous sequences were used to identify homologous linkage groups across the independent maps (Figs 4, 5 and 6 and Supplementary Table S9). Comparing our integrated map to the previously published *L*. *vannamei* SNP map from Du, *et al*.^3^, 29 linkage groups were able to be matched. Marker order was highly conserved (Figs 4 and 7), although, six SNPs were placed on alternative linkage groups. Within this comparison, our integrated map indicates that LG11 and LG34 from Du, *et al*.^3^ may be able to be merged due to common SNPs on LG14 in the present map. In addition, the map produced in this study indicates that LG1 of Du, *et al*.^3^ may be a concatenation of two separate linkage groups. A total of 35 linkage groups from Yu, *et al*.^2^ were able to be matched to the linkage groups within the present integrated map (Fig. 5). Within the 67 common SNPs between these two maps, only two SNPs were placed on alternative linkage groups. However, homologous sequence matching indicates that LG7 and LG17, as well as LG26 and LG29 may be merged due to being mapped to LG35 and LG39 respectively in the present map. Comparisons to Baranski, *et al*.^45^ revealed 44 linkage groups matched by homologous sequences (Fig. 6). Marker synteny was highly conserved with only three markers being assigned to alternatively matched linkage groups (Figs 6 and 7). Both LG39 and LG43 from Baranski, *et al*.^45^ were matched to SNPs placed within LG2 reported here, which were placed with high significance (two-point LODs ranging from 10.8–39.6), indicating a potential merger.

## Discussion

The rapid advancement of genetic and genomic technologies has made the generation of genome-wide genomic resources available for many non-model species. Genome-wide markers with known gene function and position are highly useful in comparative mapping studies, genome-wide association studies, and linking gene function to traits of interest. By using the high-throughput sequencing approach, this study provides over 234,452 putative *in-silico* SNPs and 26,662 filtered high-quality SNPs (MAF ≥ 0.25, read depth ≥ 10). A total of 8,967 high-utility SNPs were incorporated into a commercial array allowing cost-effective routine genotyping of validated content. In addition, 4,817 of these SNPs were placed within a moderate density integrated linkage and LODE map allowing insights into the genome structure of *L*. *vannamei* and comparisons to previously published genome maps in penaeids. These resources vastly improve the publically available genomic resources available for this important commercial species and have high-utility in studies aiming to identify genomic regions linked to traits of interest. Furthermore, the current integrated genetic map will help with forthcoming *L*. *vannamei* genome sequence assemblies, by providing robust gene-associated reference maps to anchor and orientate sequence data.

Highly reliable genotypes are integral to ensure the generation of integrated genetic maps and genome association studies are accurate. The success of Type I SNP assay development can be attributed to the quality of the EST sequence data used, sequence depth, *in-silico* MAF cutoff and SNP flanking region composition^51, 52^. By utilising a SNP mining approach within 25 Gb of assembled transcriptome-wide sequence data (~10× genome coverage), and applying strict SNP discovery filtering parameters (i.e. MAF of 0.25, read depth > 10, a minimum of two minor allele reads, a minimum flanking sequence quality of 25, and no observed variation in the flanking probe design region), we ensured that the SNPs reported within this study are of high utility and are dispersed throughout the genome. In addition, since all SNPs reported here were designed within large expressed contigs (N50 of 2,375 and average contig length of 1,429 bp) which are generally well conserved, they are not only known to be associated with functional genes, but will also be highly useful in ongoing comparative genomic analyses and genome sequence assemblies.

Standard measures of quantifying the success of genotyping arrays are the conversion (the proportion of SNPs producing genotypes) and validation (the proportion of SNPs that are polymorphic in a population) rates. Conversion and validation rates observed within this study (i.e. 80.95% and 95.62% respectively) were relatively high compared to Illumina genotyping arrays designed on other aquaculture species. Previous conversion and validation rate of Illumina panels from aquaculture species such as the silver-lipped pearl oyster (*Pinctada maxima*), the pacific oyster (*Crassostrea gigas*), European flat oyster (*Ostrea edulis*), rainbow trout (*Oncorhynchus mykiss*), Atlantic salmon (*Salmo salar*) and catfish (*Ictalurus punctatus*) range from 70.3–92.0% and 48.0–59.9% respectively^26, 53–57^. In comparison, the current *L*. *vannamei* array is very similar to the well-established Illumina livestock arrays such as the chicken, goat, bovine, porcine and the domestic horse which have conversation and validation rate that range from 88–97.5% and 78–99.1% respectively^58–62^. The current array was validated on a large number of samples distributed throughout the most prominent industry domesticated lines. This ensures that a high proportion of SNPs included on the commercial array will be polymorphic within the majority of farmed and wild populations of *L*. *vannamei* worldwide.

With the advent of genotype by sequencing (GBS) approaches for generating genotypic data, there has been a move away from solid state SNP genotyping arrays^63^. However, there are significant benefits of using solid state SNP arrays over GBS. For example, the laboratory techniques and procedures required to undertake genotyping as well as integral downstream bioinformatics analysis are much simpler and require less technical knowledge. As a result, genotyping arrays usually have a much quicker turnaround and are much more robust and less prone to errors^64, 65^. In addition, SNP genotyping arrays can be automated leading to higher reproducibility. Per sample, genotyping arrays are generally more expensive than GBS approaches, however, as long as the SNP arrays were designed with loci that are polymorphic within the populations for its intended use, there are many benefits that can be yielded over any GBS approach.

Assigning gene annotation and ontology terms to SNPs provide valuable insights into the functional biology and trait architecture particularly when coupled with location information within the genome. A total of 27,477 of the 76,963 contigs utilised in SNP discovery were annotated with one or more gene ontology terms. The proportion of annotated contigs reflects the still limited amount of annotated sequence data for decapods in the public domain. The major GO terms returned including cellular, metabolic and single-organism process in Biological Process; binding and catalytic activity in Molecular Function; and cell, organelle, membrane and macromolecular complexes in Cellular Components were all reflective of the proportions of GO terms returned within previous studies within penaeid transcriptome studies^10, 66^. In addition, the GO distribution patterns of protein coding genes and therefore gene compositions between the two shrimps was reported to be similar^66^.

Out of the 6,379 SNPs deemed suitable for linkage mapping, 4,370 were successfully placed via linkage analysis. An additional 447 SNPs were placed when integrating LODE methodologies resulting in a total of 4,817 SNPs mapped. The power of placing SNPs on a linkage map comes from the number of informative meiosis events within the reference mapping families. The average number of informative meiotic event across all mapped SNPs was 147.0, compared to 28.3, for all unmapped SNPs. This significant drop in the number of informative events is a major contributor to the ability to firstly assign a SNP to a linkage group, and finally order the markers unambiguously. In addition to the placement of markers, the number of informative meiotic events adds power to teasing apart the LD blocks or binning of markers placed at the same location on the linkage map. There were a number of clusters of co-localised markers within the map presented in this study (i.e. there were 4,817 markers places within 1,752 unique locations, indicating that on average, there were 2.75 markers co-localised throughout the genome). An increase in either the number of individuals per family, or the number of families should result in higher power to refine the position of these co-localised markers. Nevertheless, considering there were only minimal evidence of sex-specific recombination, family specific recombination and segregation distortion, the assignment of the SNP markers throughout the linkage mapping procedure is considered to be highly accurate.

To assign additional orphaned markers to the linkage map that were not assigned a position within linkage analysis, a locus ordering by disequilibrium (LODE) mapping procedure was implemented as described in Khatkar, *et al*.^16^. LODE methodologies rely on the linkage disequilibrium (LD) information from unrelated samples within a population and do not require specific resource populations or mapping families. A reliable estimate of *r* ^*2*^ (statistical measure of LD) can be successfully obtained from a minimum samples size of 75 unrelated individuals^40^. Within this study, a total of 1,963 individuals (from prominent domesticated industry lines) were included in LODE analysis which allowed the generation of accurate estimates of LD. In doing so, the LODE method was able to harness additional samples and linkage disequilibrium information (outside of linkage mapping families) that was useful to place 447 additional orphan SNPs.

The estimation of LD does have some assumptions and limitations. The precision of determining locations of orphan SNPs by this method depends on the extent of linkage disequilibrium and density of the framework map. Furthermore, the extent of linkage disequilibrium, among other factors, depends on population structure. Therefore the number of LODE placed SNPs within this study may be increased by utilising larger random mating wild populations (to stabilise genetic drift).

Using the observed length of the integrated linkage and LODE map, the expected genome length of *L*. *vannamei* was calculated to be 4,619.3 cM (sex average) and the estimated genome coverage is 98.12%. This is comparable to the sex-average linkage map from Yu, *et al*.^2^, which incorporated 4,817 loci and had a total genome map length of 4,341.39 cM and genome coverage of 98.39%. Previous linkage maps to this contained fewer loci and as such are less accurate and smaller in size (i.e. 3677.65–4025.5 cM; refs 12–14).

To date, no complete shrimp genomes have been published due to their large genome size (ranging from 2.17 Gb to 2.64 Gb) and high levels of duplication^2, 66^. In addition, previous comparative mapping in penaeid shrimps has been very limited, although, some comparative mapping and divergence times have been recently conducted in decapod shrimps within the Pancrustacea clade using transcriptome data^66^. By comparing homologous loci between *L*. *vannamei* and *P*. *monodon*, this study reports one of the first genomic comparisons for gene order and synteny within penaeids. Homology was investigated in two *L*. *vannamei* maps^2, 3^ and one *P*. *monodon* linkage map^45^.

Within the homologous loci between the *L*. *vannamei* linkage map published in Du, *et al*.^3^ and the integrated map within this study, the gene order is well conserved. In total, only six out of the 83 homologous loci were assigned to alternative linkage groups). Positional two-point LOD thresholds of five of the six SNPs calculated from the current map ranged from 4.5 to 27.1 (informative meiotic events ranging from 98 to 230) indicating high statistical support for their current assignments. Only one marker, LV1007 had a low two-point LOD placement threshold of 0 resultant from 70 informative meiotic events. With a higher density of markers and number of informative meiotic events in the current integrated map, the placement of the large majority of SNPs is expected to be more accurate. Similarly, only two out of the 67 common SNPs were assigned to alternative linkage groups when comparing the map presented in this study to the *L*. *vannamei* map published in Yu, *et al*.^2^. These two SNPs had high positional two-point LOD thresholds at 14.4 and 19.3, indicating strong support for their placement in the current map.

The total length of the female and male maps for *P*. *monodon* map were 4,060 cM and 2,917 cM respectively. Within *P*. *monodon*, the female map was 28% larger than the male map indicating greater recombination frequency in female over males. Even though large differences in recombination rate between sexes have been reported in other penaeids^12, 13, 45, 67–69^, it was not observed within the *L*. *vannamei* map reported in this study (female and male map length of 4,530.6 cM and 4,522.3 cM respectively). Sex-specific recombination is highly variable throughout existing reported penaeid maps, which may come down to the number of markers mapped to the respective sex maps, whereby various numbers of markers mapped for respective sexes could be influencing the recombination rates observed. The incorporation of many more markers, families and offspring (and therefore more total informative meiosis events) within the integrated map is expected to produce a more accurate estimation of sex-specific recombination rate than the existing maps.

*P*. *monodon* and *L*. *vannamei* share an identical chromosome number of 2n = 44^13, 67^. A comparison of the 44 LGs from our integrated map to the *P*. *monodon* map produced in Baranski, *et al*.^45^ returned substantial macrosynteny throughout the 275 homologous loci identified despite their estimated divergence of 95 million years ago^66^. Only three SNPs were assigned to alternative linkage groups, whereby the two-point LOD SNP placements for the three SNPs placed on different linkage groups ranged from 6.6 to 22.3 (with informative meiotic events ranging from 79 to 226). There is minor evidence of interchromosomal rearrangements and marker shuffling between the species [i.e. within linkage group pairs LG6 (*L*. *vannamei*) and LG14 (*P*. *monodon*); LG40 (*L*. *vannamei*) and LG22 (*P*. *monodon*); LG34 (*L*. *vannamei*) and LG41 (*P*. *monodon*); and LG15 (*L*. *vannamei*) and LG44 (*P*. *monodon*)], however, comparisons between a higher density of markers is required before inferences of chromosomal rearrangements can be made reliably. Overall, the robustness of the marker orders in all maps is demonstrated by the high correlations between marker orders across most linkage groups calculated from independent analysis. The sporadic marker disagreements may be due to either genotyping errors, or differences in the mapping algorithms used during map construction^1^.

Penaeid genomics has come a long way in the last 15 years. Many species now have significant genomic resources which will enable more advanced methods of breeding such as marker assisted and genomic selection^70^. These novel techniques may help increase disease resistance to specific emerging diseases which is a major priority for current shrimp breeding programs. It is predicted from simulated genetic advancement using genomic information in selection programs for survival and disease resistance was up to 2.6 times as effective than that of phenotypic sib-selection alone^70^. Furthermore, considering vaccination is not an option and management interventions to curve disease are usually unfeasible, several shrimp breeding programs have already been implemented in a number of countries to improve disease resistance as reviewed in Neira^71^, Rye^72^, and Castillo-Juárez, *et al*.^70^. With the continuing development of genomic resources in penaeids, incorporation of genomic information into breeding programs is a viable option promising to increase the accuracy of selection and therefore response compared to conventional selection^70^.

Developing a large set of type I genome-wide molecular markers and genomic maps for *L*. *vannamei* is a fundamental step towards further understanding the genomic structure and genetic contribution to commercially important traits. The development and validation of a large EST-derived SNP database and commercial genotyping array as described within this study will expedite the development and incorporation of genomic information into advanced selective breeding programs by enabling researchers to cost effectively genotype a large number of individuals within breeding programs. In addition, this study provides an integrated linkage and LODE map for *L*. *vannamei*, which revealed high macrosynteny between *L*. *vannamei* and *P*. *monodon* with only a small number of occurrences of inter chromosomal rearrangements. Combined, these data provide an important resource for genetic association studies, comparative genomics, and assisting in genome assemblies across several related shrimp taxa.

## Electronic supplementary material

Supplementary Information

Supplementary Table S1

Supplementary Table S3

Supplementary Table S4

Supplementary Table S5

Supplementary Table S7

Supplementary Table S8

Supplementary Table S9
