# Supplementary material for: A comparative integrated gene-based linkage and locus ordering by linkage disequilibrium map for the Pacific white shrimp, Litopenaeus vannamei
Source: Sci Rep. 2017 Sep 4;7:10360. doi: 10.1038/s41598-017-10515-7 (PMC5583237; doi:10.1038/s41598-017-10515-7)

A comparative integrated gene-based linkage and locus ordering by linkage disequilibrium map for the Pacific white shrimp, *Litopenaeus vannamei*

David B. Jones[1*], Dean R. Jerry[1,2], Mehar S. Khatkar[2,3], Herman W. Raadsma[2,3], Hein van der Steen[4], Jeffrey Prochaska[4,#a] Sylvain Forêt[5] and Kyall R. Zenger[1,2]

[1] *Centre for Sustainable Tropical Fisheries & Aquaculture, and the College of Science and Engineering, James Cook University, Townsville QLD, Australia.*

[2] *ARC Hub for Advanced Prawn Breeding, James Cook University, Townsville QLD, Australia.*

[3] *Sydney School of Veterinary Science, The University of Sydney, Camden, NSW, Australia.*

[4] *Global Gen, Desa Cikiwul Bantar Gebang Bekasi, Indonesia.*

[5] *ARC Centre of Excellence for Coral Reef Studies, James Cook University, Townsville, Queensland, Australia*

[#a] *Current Address: Amity Aquaculture, LLC. Cheyenne, WY, USA*

* Corresponding author:

Email: david.jones3@jcu.edu.au (DBJ)

Supplementary Information

Supplementary Table S1. Descriptive statistics for all SNPs included on the custom *L. vannamei* Illumina 10k iSelect BeadChip. Statistics are reported for *in-silico* design (including source sequences) and SNP performance at genotyping (based on individually genotyped *L. vannamei* DNA). File format: xls

Supplementary Methods. Parental genotype reconstruction using allele frequency pools via the $\hat{p}_{n3}$ method. File format: pdf

Supplementary Fig. S2. Pedigree structure of the linkage mapping families. Circles represent dams, squares represent sires and diamonds represent individual families. File format: pdf

Supplementary Table S3. Grandmaternal and grandpaternal reference mapping families selected for linkage mapping analysis. File format: xls

Supplementary Table S4. Blast2GO results of all unique contigs. Sequence descriptions from Blast results, GO annotations, enzyme codes and InterPro IDs are reported. File format: xls

Supplementary Table S5. Detailed statistics on all 6,379 phased SNPs suitable for mapping analysis. The source sequence from which the SNPs were designed is reported along with minor allele frequency and sequence length. All SNPs were assigned a destination of either 'Uninformative', not returning sufficient informative meiosis within the mapping families to be mapped; 'Not assigned to LG', returned informative meiosis but was not included in a LG; 'Assigned to LG but not mapped', SNPs clustered during initial mapping but could not be positioned; 'Mapped', SNPs which appear in the final comprehensive map; or finally 'Placed by LODE', SNPs positioned using LODE analysis. Linkage groups, positions, informative meiosis and two-point (2pt) LOD placement cutoffs are listed

along with the Haldane and Kosambi cM for the sex average, female and male maps are all reported. File format: xls

Supplementary Fig. S6. Cumulative cM plots for the sex average, female and male maps across all 44 linkage groups. File format: pdf

Supplementary Table S7. Significant segregation distortions throughout mapping families. File format: xls

Supplementary Table S8. The extent of LD per linkage group and across the genome. File format: xls

Supplementary Table S9. Comparative mapping annotations for the integrated map, Baranski, et al. [45], Du, et al. [3] and Yu, et al. [2]. Data from Yu, et al. [2] and Baranski, et al. [45] was utilised under a Creative Commons Attribution 4.0 International License (https://creativecommons.org/licenses/by/4.0/). File format: xls

Supplementary Methods: Parental genotype reconstruction using allele frequency pools via the $\hat{p}_{n3}$ method.

To reduce the number of samples to be run on the genotyping array platform, full genotypes of unknown or ungenotyped parents were reconstructed using two methods. Firstly, a known parental genotype and nauplii pool allele frequencies were utilised to reconstruct missing parent genotypes. Secondly, the known parent and individual offspring genotypes were utilised to reconstruct the unknown parental genotypes. In total, 653 parental genotypes (641 via nauplii pools and 12 via individually genotyped family offspring) were successfully reconstructed.

For the first approach, SNP genotype data for all nauplii pool samples were derived from raw intensity values of the X and Y alleles. For individuals, genotype data such as AA/AB/BB were also provided. Normalised allele frequencies from pools of genomic DNA were estimated using the method Normalization 3 ($\hat{p}_{n3}$) as described in Peiris *et al.* (2010). The equation is as follows;

$$(4) \qquad \hat{p}_{n3} = \left\{ \begin{array}{ll} \frac{1}{2}\left(\frac{\hat{p}_k - \bar{\hat{p}}_{k_{BB}}}{\frac{1}{2} - \bar{\hat{p}}_{k_{BB}}}\right), & \hat{p}_k \le \frac{1}{2}, \\ 1 - \left(\frac{1}{2}\left(\frac{\hat{p}_k - \hat{p}_{k_{AA}}}{\frac{1}{2} - \hat{p}_{k_{AA}}}\right)\right), & \hat{p}_k > \frac{1}{2}, \end{array} \right\}$$

$$\hat{k} = \bar{X} \,/\, \bar{Y},$$

$$\hat{p}_k = X \,/\, (X + \hat{k}Y)$$

where $\hat{p}_k$ is heterozygote-corrected frequency estimation; k (the heterozygote correction factor) is the ratio of the X and Y intensities of heterozygotes; $\bar{X}$ and $\bar{Y}$ are the average of X and the average of Y of known heterozygous (AB) individuals for each SNP. The correlation between parentage genotypes and predicted allelic frequencies of progeny DNA pools is the sum frequency of any allele from DAM and SIRE at one SNP will be equal to the predicted allelic frequency (pn3) of their progeny DNA pools at the same SNP. Since each SNP has only two alleles (A/B), the allelic frequency of individually progeny will be placed into one of five value bins (0, 0.25, 0.5, 0.75 and 1).

SNP data from 1,327 individual white-leg shrimp samples were utilised to calculate the heterozygote-corrected frequency estimation ($\hat{p}_k$) and the heterozygote correction factor (k) for each of the 6,379 SNPs. A total of 22 family allele pools had array genotype data from both of their parents and were used as positive controls. Reconstructed parental genotypes were compared to genotypes produced on the array to evaluate the success of genotype reconstruction. SNPs with more than one incorrect predicted genotype (deemed unreliable SNPs) were excluded to gain the successful inferred sire genotyping rate of ~ 99% over these 6,379 SNPs. Following this, SNP data of 678 DNA nauplii pools of progeny and their known Dam's genotypes were used to predict the missing genotypes of sires.

Supplementary Fig. S2

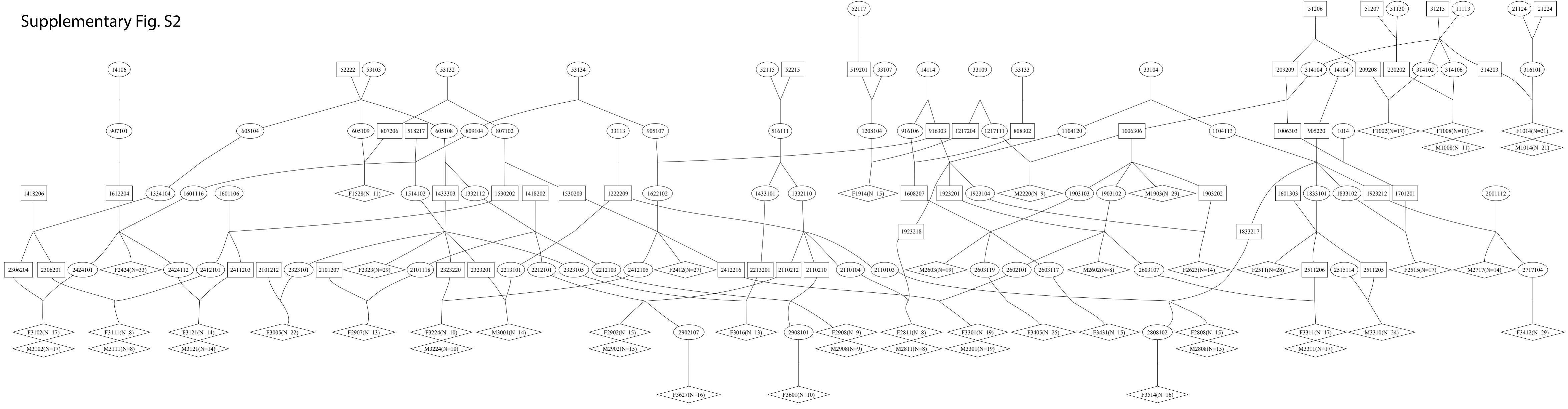

Supplementary Fig. S6

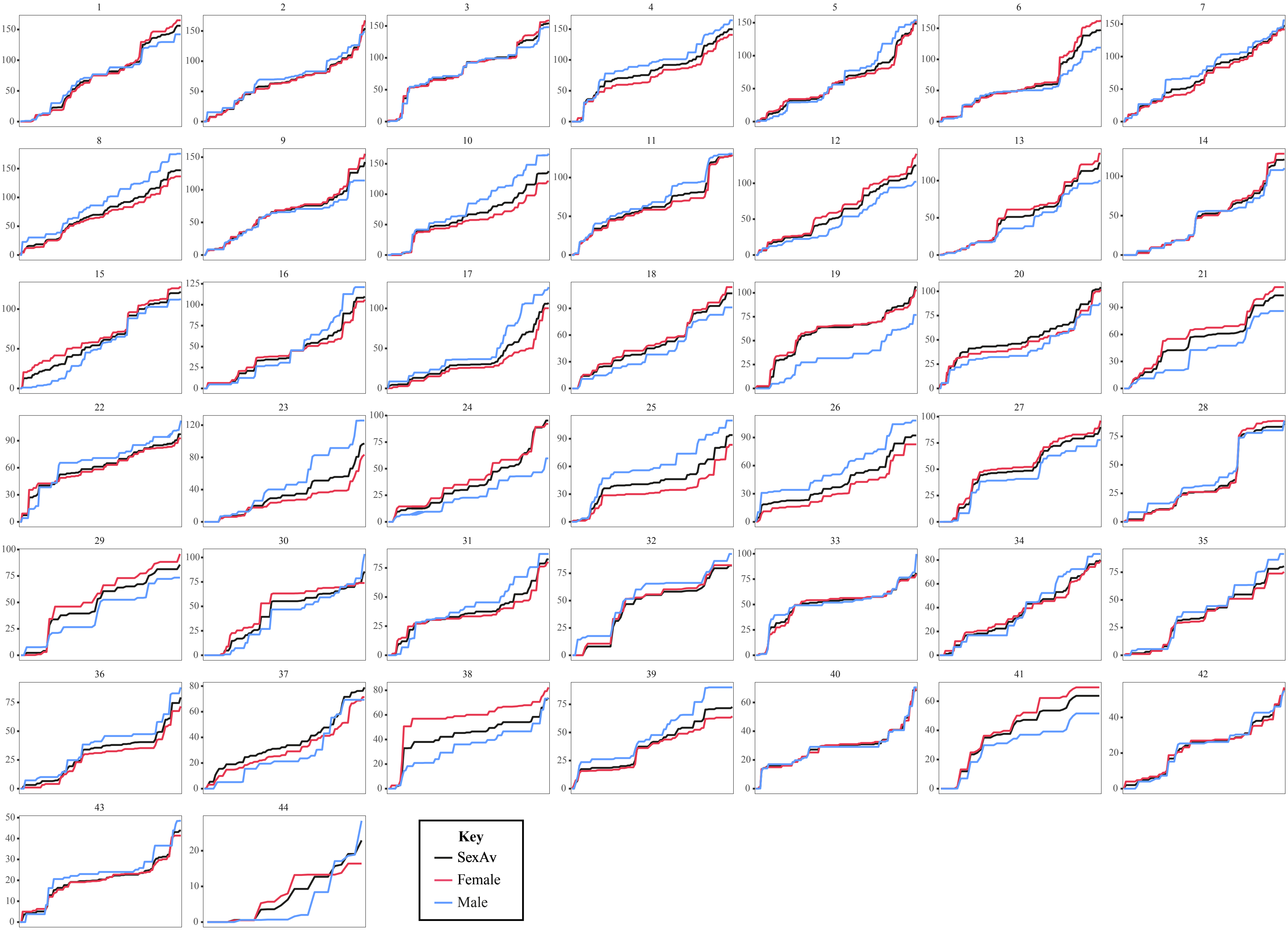

Supplement: Supplementary file 1 — Supplementary Information [file 41598_2017_10515_MOESM1_ESM.pdf]
